# Network propagation in the cytoscape cyberinfrastructure

**DOI:** 10.1371/journal.pcbi.1005598

**Published:** 2017-10-12

**Authors:** Daniel E. Carlin, Barry Demchak, Dexter Pratt, Eric Sage, Trey Ideker

**Affiliations:** Department of Medicine, University of California-San Diego, San Diego, California, United States of America; University of Southern California, UNITED STATES

## Abstract

Network propagation is an important and widely used algorithm in systems biology, with applications in protein function prediction, disease gene prioritization, and patient stratification. However, up to this point it has required significant expertise to run. Here we extend the popular network analysis program Cytoscape to perform network propagation as an integrated function. Such integration greatly increases the access to network propagation by putting it in the hands of biologists and linking it to the many other types of network analysis and visualization available through Cytoscape. We demonstrate the power and utility of the algorithm by identifying mutations conferring resistance to Vemurafenib.

“This is a *PLOS Computational Biology* Software paper.”

## Introduction

Network propagation has become a critical analysis technique in computational systems biology. Most commonly, the basic network propagation process starts with some query nodes, and a network. The query nodes are given some initial value, then a smoothing process is applied to the initial values on those query nodes, passing some of the value to neighboring nodes. By examining the final distribution of values, we can, for instance, find a subnetwork where the nodes are closely related by the network to the original query. More abstractly, network propagation supplies a robust estimate of network closeness, which can be used for many different applications. In computational biology, the most common meaning for the nodes is genes, and the edges usually represent various types of functional relationships between genes, for instance protein binding interaction, transcriptional regulation or signaling by phosphorylation.

Of the many applications of network propagation in systems biology, one of the most critical has been disease gene prioritization in genome-wide association studies. Genetic variants or mutations that otherwise would not pass a test for statistical significance, due to low power of association with the disease phenotype, can be prioritized due to their close association with each other in molecular networks. Koehler et al. [1] were some of the first to use network propagation in this fashion, which has since been extended to prioritizing protein complexes [2] and applied in a variety of other disease settings [3,4].

Vandin et al. [5] applied network propagation to discover significantly mutated subnetworks in cancer. Since then, the method has become a cornerstone of cancer genome analysis. Hofree et al. [6] used a network propagation method (Network-Based Stratification, or NBS) to cluster cancer patient data, Paull et al. [7] identified causal paths linking mutations to expression regulators, and Liu et al. and Zhong et al. [8,9] applied the NBS method across a wide variety of cancer types.

Given the success of this wide variety of applications, it is essential for network propagation to be readily available to any biologist, including those without dedicated bioinformatics support. Cytoscape is a well-established platform for many network applications [10], so it is a natural choice to host a network propagation algorithm. There have been a few Cytoscape Apps (GeneMania [11], propagate, and TieDIE) that have previously implemented network propagation algorithms; however, in these cases the basic network propagation operation is embedded within a more complex and extensive workflow or does not scale well to large biological networks. Nonetheless, the presence of these Apps demonstrate the community’s need for a stand-alone, robust network propagation algorithm.

Here we introduce Diffusion, a network propagation algorithm delivered as both a new Cytoscape feature and as an internet service available to a broad array of web, desktop and server-based applications such as Jupyter notebooks. The service is callable via a REST-based Application Programming Interface [12,13] (API, documented at https://github.com/idekerlab/heat-diffusion).

### Design and implementation

We chose to implement Diffusion as a service to leverage the myriad benefits of Service Oriented Architectures (SOAs) [13]. In an SOA design, computations are packaged as components, called services, running on servers that are remotely accessible across the Internet. Consequently, client applications (regardless of operating system or environment) can call the service without needing to install it on local hardware or provision substantial memory or processor resources. Additionally, the SOA enables Diffusion to produce consistent results for all clients while allowing transparent and seamless service improvements and optimizations.

Diffusion is hosted on servers operated by the National Resource for Network Biology (nrnb.org), though the code, which is Open Source, can be hosted elsewhere, too. Additionally, it is part of the Cytoscape Cyberinfrastructure (CI), a collection of services that deliver functionality on networks encoded by the common transfer format called CX. Biological applications that use Diffusion are also well positioned to compose multiple CI services to create novel and complex workflows quickly.

The Cytoscape implementation of Diffusion provides a simple and convenient user interface that allows a user to visually select a query node set, invokes the Diffusion service, and then visualizes the diffusion results. It is delivered as a core feature as of Cytoscape v3.6, and it is available as an app (http://apps.cytoscape.org/apps/Diffusion) for previous Cytoscape versions.

The concept of network propagation is commonly implemented by either of two related algorithms. The first algorithm goes by several names: Google Pagerank, the random surfer model, or random walk with restart. The second algorithm is called heat diffusion. The difference between these two approaches is a modeling choice, and each has proven competitive in different applications. In fact, random walk with restart is equivalent to heat diffusion with the following assumptions; no restart, undirected edges on the graph, time steps that approach zero in length, and not running the random walk to equilibrium, but rather stopping after some short amount of time. In the random walk case, the amount of spread from the original distribution is parameterized by the restart probability, while in heat diffusion the spread is controlled by the time parameter t below. However, due to algorithmic advantages in memory use [14] the heat diffusion model is currently much faster to compute, and so we have selected it here. The calculation is given by:

d=h*exp⁡(−Lt) (Eq 1)

where h is a vector representing the original query, and d is the result vector. L is the graph Laplacian defined by D − A, where D is a diagonal matrix holding the degree of each node and A is the graph adjacency matrix of the input network. The scalar parameter t is the total time of diffusion, which controls the extent to which the original signal is allowed to spread over the network. We use a default value of t = 0.1. The expression exp(*) is the matrix exponential.

The probabilistic interpretation for this calculation is that if the input values are a probability distribution of starting positions for particles diffusing across the edges of the graph, the final value is the position distribution after t time units. As t goes to infinity, the probability distribution approaches a uniform distribution over all nodes.

We have included advanced settings to adjust the time parameter. However, we have found that the order of nodes that is returned by a query is quite robust to the choice of t. In order to demonstrate this, created a scale-free random graph of increasing size and diffused a random query of 10 percent of the nodes. S1 Fig shows the Spearman correlation between adjacent time steps for various sizes of network and S2 Fig shows the same for a fixed network of 10,000 nodes with various proportions of the graph included in the query. The fact that all sizes and proportions of query nodes reach a very high degree of correlation near t = 0.1 indicates that the order of nodes does not change much around the default choice of this parameter, regardless of the nature of the network and query.

We have implemented this calculation using the scipy python library [15] on our servers. However, the installation of scipy is not necessary for the use of the service. Alternatively, this same queries can be run programmatically through the API. For an example, see the Jupyter Notebook at: https://github.com/idekerlab/heat-diffusion/blob/master/demo.ipynb.

## Results

**Fig 1:**
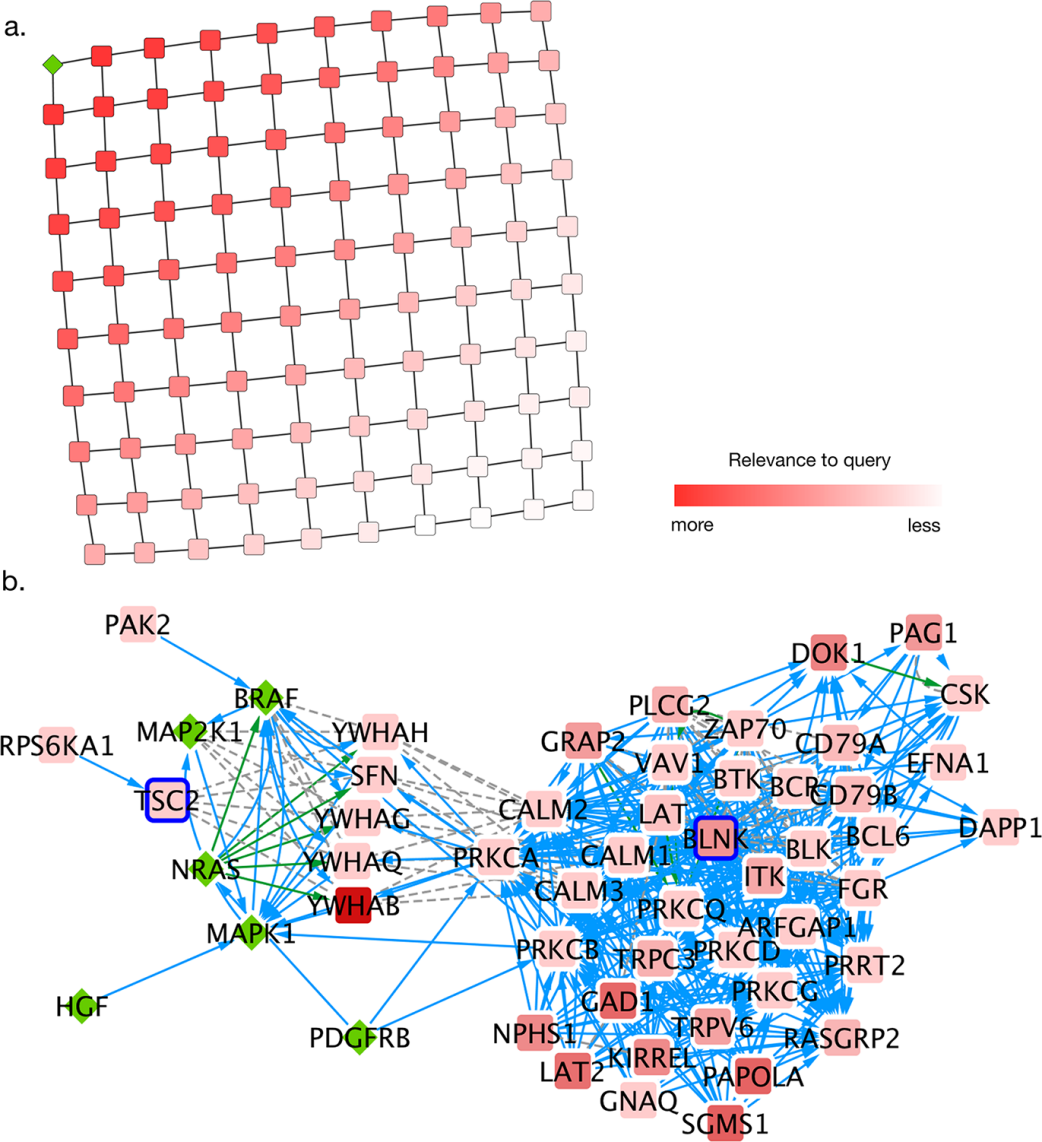
Example uses of diffusion. a. Diffusion of heat from a single query gene in the upper left, denoted with a green diamond, demonstrates that heat diffusion recapitulates network distance to the query set. b. A local network hypothesis for Vemurafenib resistance in cell line LOX-IMVI, correcting for a responsive cell line, A375. LOX-IMVI mutated genes appear outlined in red. The original Vemurafenib query genes appear as green diamonds. Three interaction types are present in this network: Grey dotted lines are protein-protein binding interactions, green arrows indicate control of localization, and blue arrows indicate phosphorylation.

To demonstrate the Diffusion service with a simple example, we created a 10-by-10 grid network and diffused a query from the upper left corner of the grid. The resultant output (Fig 1A) is a good proxy for distance to the upper left corner.

As a biological example, we chose to investigate a Vemurafenib resistant melanoma cell line, LOX-IMVI, as compared to a sensitive cell line A375 [16,17] with the goal of implicating mutations that may be affecting LOX-IMVI’s response to the drug. As an input query, we chose six genes (BRAF, PDGFRB, NRAS, HGF, MAP2K1, MAPK1) with known relationships to the drug [18–20]. The goal of this analysis is to find a subnetwork that is near the mutations of LOX-IMVI (which may be conferring resistance) and the known drug associated genes, but not near mutations that occur in the sensitive A375 line. For the underlying network, we used the NCI Pathway Interaction Database [21], an amalgamation of expert-curated cancer pathways. The version of this database that we used is provided in the supporting information as S1 File. As described in the following protocol, using a cutoff of the top 10 percent of nodes relevant to both Vemurafenib and LOX-IMVI mutations, but not A375 mutations according to diffusion, we produced a subnetwork of 53 nodes and 448 edges (Fig 1B). We demonstrate how this network-based hypothesis was reached using our Cytoscape application in the following example:

First, we performed a simple query to create a subnetwork relevant to Vemurafenib, based on the literature-implicated genes above:

1. Download and install Cytoscape from http://www.cytoscape.org/

2. Start Cytoscape, and instantiate a new session.

3. Install the “diffusion” app. **Apps>App Manager> Select “diffusion” > Click install** (Cytoscape versions earlier than 3.6).

4. Import the example network. **File>Import>Network>File**. In this case, S1 File.

Select a set of nodes using the mouse or by entering the node names separated by spaces into the Search Bar. We used BRAF, PDGFRB, NRAS, HGF, MAP2K1, and MAPK1. This will select the nodes with these gene names.

5. Right click on one of the selected nodes and select **Diffusion>Diffuse Selected Nodes**. This creates three table columns:

a. diffusion_input is a column representing the selected nodes. This is the h vector in Eq 1

b. diffusion_output_heat is a column representing the output vector d in the Eq 1

c. diffusion_output_rank is the rank of diffusion_output_heat from largest to smallest.

**Fig 2:**
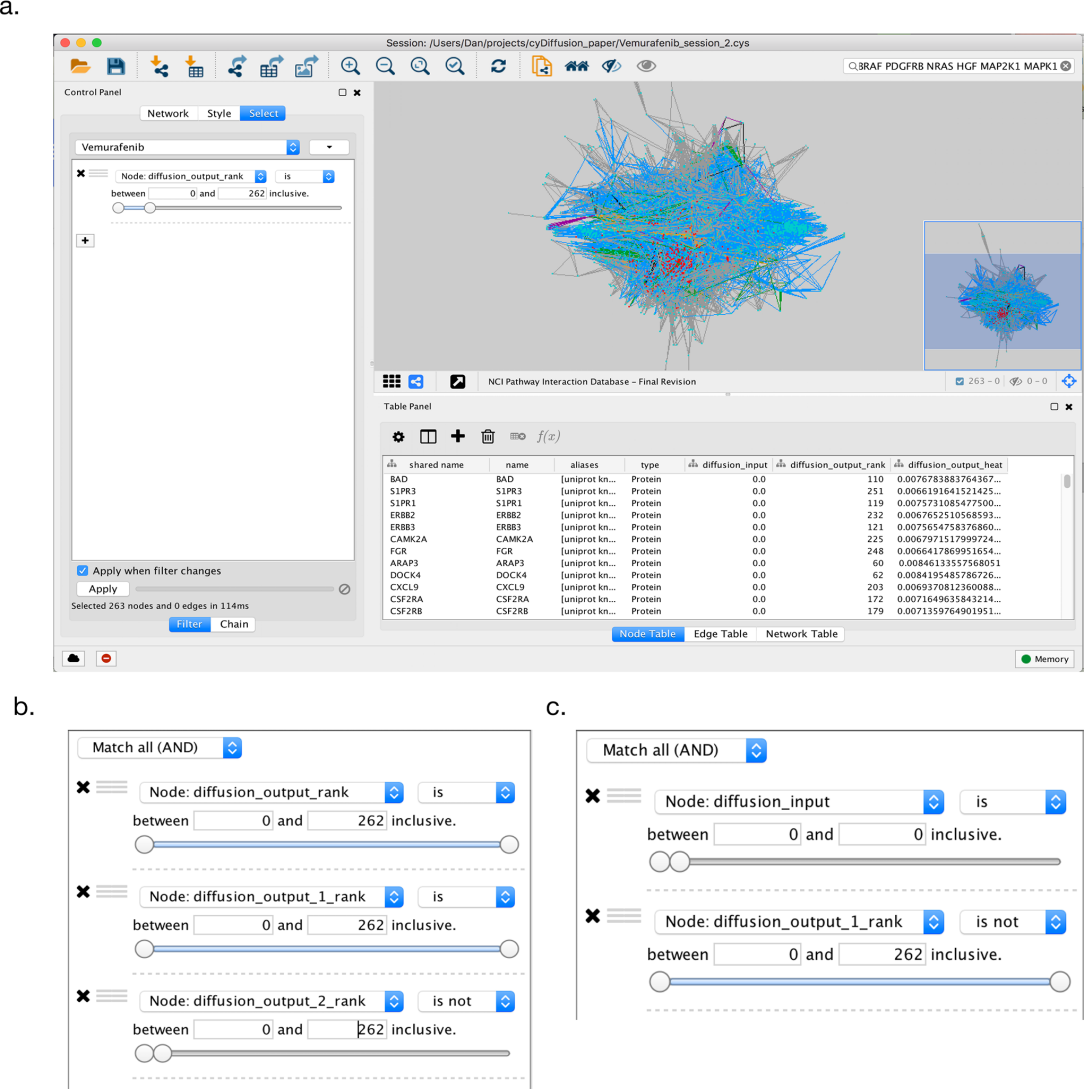
Cytoscape example screenshots. a. Cytoscape after a simple diffusion query. The selection filter is set to pick the top 10 percent most relevant genes by rank to the input query. b. The filter settings for the selection of the subnetwork that is closely related to the Vemurafenib genes and the mutations in the LOX-IMVI cell line, but not the A375 genes. c. The filter settings for the original the query’s first neighbors that were not in the result network from step 15.

6. Construct a filter to select the most relevant subnetwork. First click on Select on the Control Panel on the left. Click the plus sign to create a new selection criterion. Choose Column Filter to specify that the information for the filter is in a Node Column. From the Choose column selector menu, select Node:diffusion_output_rank. Then select the most relevant nodes by sliding upper rank threshold slide to the left to select the desired number of nodes. In our case, we selected the top 10 percent most relevant genes, i.e. where the rank of the heat is less than 262 (Fig 2A).

7. Click the **File>New>Network>From selected nodes, all edges** to create a new network from the most relevant subnetwork.

Subsequent queries after the first diffusion will create new input and output columns, with each new entry denoted with an incremented counter. One can also use an imported table of data to drive a query. We have provided the mutations of the two cell lines in question (LOX-IMVI and A375) in a table that can be imported, downloaded from the Cancer Cell Line Encyclopedia [22]. Steps 9–12 provide an example of performing diffusion based on an imported set of features from a table:

9. Return to the original network by clicking on the Network tab from the Control Panel and selecting the original network.

10. Import the data table by choosing **File>Import>Table>File** and selecting the data table, S1 Dataset.txt. Click OK to import the data table as node features.

11. Create a new selection filter based on the imported data by clicking the Select tab on the control panel, then clicking the down arrow next to the filter name. Select Create new filter and name the filter. Click the plus sign to create a new selection criterion. Choose Column Filter to specify that the information for the filter is in a Node Column. From the Choose column selector menu, select one of the node columns corresponding to the data that you imported from the table, in this case LOXIMVI_SKIN. Slide the selector so that only nodes with a 1 in the relevant node column are selected.

12. Right click on one of the selected nodes, and select **Diffusion>Diffuse Selected Nodes**. Since this is the second query on this network, two new diffusion result columns are added to the network, named diffusion_output_rank_1 and diffusion_output_heat_1. Subsequent diffusion queries after the first are denoted with incremented integers in the output column names.

Diffusion results can be combined in informative ways. In steps 13–15 we combine our previous two query results with a third query from the A375 mutations to create a final network. The goal of this third criteria is to select against mutations that are similar to observed mutations in the Vemurafenib sensitive A375 cell line, which may be unlikely to be conferring resistance in LOX-IMVI.

13. Repeat steps 11 and 12 with the A375_SKIN column to create a new diffusion result.

14. Create a new filter, selecting nodes that are in the top 10 percent of each of the original Vemurafinib query and LOX-IMVI query, but not in the top 10 percent of the A375 query (Fig 2B).

15. Invoke **File>New>Network>From selected nodes, all edges** to create a new network.

Although not strictly necessary, it can be useful to add in the original query to better understand why nodes were selected. In the following sequence, we add in the original Vemurafenib nodes.

16. With the original complete NCI-PID network selected, run the original query again to select BRAF, PDGFRB, NRAS, HGF, MAP2K1, and MAPK1. Right click one of the selected nodes and click Select First Neighbors.

17. Create a new network by using **File>New>From Selected Nodes, All edges**.

18. Merge the networks created in step 15 and 17, by clicking **Tools > Merge Networks** and selecting those two networks to create a new Merged Network.

19. Filter out the first neighbors of the Vemurafenib query that did not make the LOX-IMVI filter cut by making a filter to select nodes where diffusion_input is 0 (i.e. not in the query) and diffusion_output_rank_1 is not less that 262 (Fig 2C).

20. Delete the selected nodes by clicking **Edit> Delete selected nodes**.

This technique is similar to Paull et al. [7] and Drake et. al [23], in that it uses multiple diffusion queries to select a specific subnetwork from multiple sources of evidence. TSC2 and BLNK were both mutated in the LOX-IMVI cell line and interacted with both BRAF and MAPK1, but similar mutations did not occur in A375, suggesting that these mutations may have a role in the cell line’s resistance to Vemurafenib. TSC2 is especially well supported, since it is a member of the PI3K/AKT/mTOR pathway, which has been shown to harbor resistance-conferring alterations [24].

### Availability and future directions

Future additions, such as the random walk with reset implementation, can be added to this service without any additional effort on the part of the user beyond an update of the application. We will also be adding support for real values, rather than binary inputs for the input vector, which will allow users to experiment with diffusing effect sizes on networks. This might be useful in, for instance, network-based Genome Wide Association Studies as a way to implicate weak associations that occur together in network neighborhoods. We will also be adding support for weighted edges. Edge weights may be a good way to encode interaction confidence. Different edge weights might also be an appropriate way of encoding different edge types into the same network diffusion process.

Future work on network propagation will work to bridge the gap to clinical applications; for example, considering propagated mutation profiles of patients, rather than cell lines as we have demonstrated here, will allow for the extraction of clinically relevant subnetworks that may inform patient care. In addition, many important questions remain about the use of network propagation: For instance, what are the best networks to use in different applications? How should heterogeneous edge types affect how propagation should be performed? A consistent algorithm such as the one provided here will be essential in answering these questions.

The Diffusion codebase and API documentation is available at https://github.com/idekerlab/heat-diffusion. However, this code is not required to use the service, as the API can be accessed by any script at v3.heat-diffusion.cytoscape.io. The Cytoscape plugin can be accessed through the Cytoscape Store at http://apps.cytoscape.org/apps/Diffusion, or it can be installed through the Cytoscape Application Manager. In Cytoscape Version 3.6, installation of the diffusion application is not required, as it is included in the core functionality of Cytoscape.

## Supporting information

S1 Fig Network size and time. Effect of different choices of time parameter t on the convergence of queries on different sizes of random networks. As a measure of convergence, we track the Spearman correlation between adjacent time points. Note that near the default parameter of t = 0.1, queries on all sizes of network have converged.
(PNG)

S2 Fig Query size and time. Effect of different choices of time parameter t on the convergence of queries of different proportions of a 10,000 node random network. As a measure of convergence, we track the Spearman correlation between adjacent time points. Note that near the default parameter of t = 0.1, all sizes of query have converged.
(PNG)

S1 File NCI-PID cytoscape session. This file contains the National Cancer Institute’s Pathway Interaction Database network used in the worked example.
(CX) Click here for additional data file.

S1 Dataset Cell line mutations. This dataset contains the mutations in the cell lines used in the worked example.
(TXT)

## References

[1] Köhler S, Bauer S, Horn D, Robinson PN. Walking the interactome for prioritization of candidate disease genes. Am J Hum Genet. 2008;82: 949–958. doi: 10.1016/j.ajhg.2008.02.013 18371930 10.1016/j.ajhg.2008.02.013 PMC2427257

[2] Vanunu O, Magger O, Ruppin E, Shlomi T, Sharan R. Associating Genes and Protein Complexes with Disease via Network Propagation. PLoS Comput Biol. Public Library of Science; 2010;6: e1000641 doi: 10.1371/journal.pcbi.1000641 20090828 10.1371/journal.pcbi.1000641 PMC2797085

[3] Qian Y, Besenbacher S, Mailund T, Schierup MH. Identifying disease associated genes by network propagation. BMC Syst Biol. 2014;8: S6. 10.1186/1752-0509-8-S1-S6 PMC4080512 24565229

[4] Lee I, Blom UM, Wang PI, Shim JE, Marcotte EM. Prioritizing candidate disease genes by network-based boosting of genome-wide association data. Genome Res. 2011;21: 1109–1121. doi: 10.1101/gr.118992.110 21536720 10.1101/gr.118992.110 PMC3129253

[5] Vandin F, Upfal E, Raphael BJ. Algorithms for detecting significantly mutated pathways in cancer. J Comput Biol. 2011;18: 507–522. doi: 10.1089/cmb.2010.0265 21385051 10.1089/cmb.2010.0265

[6] Hofree M, Shen JP, Carter H, Gross A, Ideker T. Network-based stratification of tumor mutations. Nat Methods. Nature Research; 2013;10: 1108–1115. doi: 10.1038/nmeth.2651 24037242 10.1038/nmeth.2651 PMC3866081

[7] Paull EO, Carlin DE, Niepel M, Sorger PK, Haussler D, Stuart JM. Discovering causal pathways linking genomic events to transcriptional states using Tied Diffusion Through Interacting Events (TieDIE). Bioinformatics. 2013;29: 2757–2764. doi: 10.1093/bioinformatics/btt471 23986566 10.1093/bioinformatics/btt471 PMC3799471

[8] Liu Z, Zhang S. Tumor characterization and stratification by integrated molecular profiles reveals essential pan-cancer features. BMC Genomics. 2015;16: 503 doi: 10.1186/s12864-015-1687-x 26148869 10.1186/s12864-015-1687-x PMC4491878

[9] Zhong X, Yang H, Zhao S, Shyr Y, Li B. Network-based stratification analysis of 13 major cancer types using mutations in panels of cancer genes. BMC Genomics. 2015;16: S7. 10.1186/1471-2164-16-S7-S7 PMC4474538 26099277

[10] Smoot ME, Ono K, Ruscheinski J, Wang P-L, Ideker T. Cytoscape 2.8: new features for data integration and network visualization. Bioinformatics. Oxford Univ Press; 2011;27: 431–432. doi: 10.1093/bioinformatics/btq675 21149340 10.1093/bioinformatics/btq675 PMC3031041

[11] Montojo J, Zuberi K, Rodriguez H, Kazi F, Wright G, Donaldson SL, et al GeneMANIA Cytoscape plugin: fast gene function predictions on the desktop. Bioinformatics. 2010;26: 2927–2928. doi: 10.1093/bioinformatics/btq562 20926419 10.1093/bioinformatics/btq562 PMC2971582

[12] Fielding RT. Architectural styles and the design of network-based software architectures [Internet]. University of California, Irvine 2000 Available: http://jpkc.fudan.edu.cn/picture/article/216/35/4b/22598d594e3d93239700ce79bce1/7ed3ec2a-03c2-49cb-8bf8-5a90ea42f523.pdf

[13] Fowler M, Lewis J. Microservices. Viittattu. 2014;

[14] Al-Mohy AH, Higham NJ. Computing the Action of the Matrix Exponential, with an Application to Exponential Integrators. SIAM J Sci Comput. 2011;33: 488–511.

[15] Jones E, Oliphant T, Peterson P, Others. SciPy: Open source scientific tools for Python, 2001. URL http://www.scipy.org. 2015;73: 86.

[16] Sun C, Wang L, Huang S, Guus J J, Prahallad A, Robert C, et al Reversible and adaptive resistance to BRAF(V600E) inhibition in melanoma. Nature. Nature Research; 2014;508: 118–122. doi: 10.1038/nature13121 24670642 10.1038/nature13121

[17] Beazley-Long N, Gaston K, Harper SJ, Orlando A, Bates DO. Novel mechanisms of resistance to vemurafenib in melanoma—V600E B-Raf reversion and switching VEGF-A splice isoform expression. Am J Cancer Res. 2015;5: 433–441. 25628951 PMC4300704

[18] Straussman R, Morikawa T, Shee K, Barzily-Rokni M, Qian ZR, Du J, et al Tumour micro-environment elicits innate resistance to RAF inhibitors through HGF secretion. Nature. Nature Research; 2012;487: 500–504. doi: 10.1038/nature11183 22763439 10.1038/nature11183 PMC3711467

[19] Nazarian R, Shi H, Wang Q, Kong X, Koya RC, Lee H, et al Melanomas acquire resistance to B-RAF(V600E) inhibition by RTK or N-RAS upregulation. Nature. Nature Research; 2010;468: 973–977. doi: 10.1038/nature09626 21107323 10.1038/nature09626 PMC3143360

[20] Wilson TR, Fridlyand J, Yan Y, Penuel E, Burton L, Chan E, et al Widespread potential for growth-factor-driven resistance to anticancer kinase inhibitors. Nature. 2012;487: 505–509. doi: 10.1038/nature11249 22763448 10.1038/nature11249 PMC3724525

[21] Schaefer CF, Anthony K, Krupa S, Buchoff J, Day M, Hannay T, et al PID: the Pathway Interaction Database. Nucleic Acids Res. Oxford Univ Press; 2009;37: D674–9. doi: 10.1093/nar/gkn653 18832364 10.1093/nar/gkn653 PMC2686461

[22] Barretina J, Caponigro G, Stransky N, Venkatesan K, Margolin AA, Kim S, et al The Cancer Cell Line Encyclopedia enables predictive modelling of anticancer drug sensitivity. Nature. Nature Research; 2012;483: 603–607. doi: 10.1038/nature11003 22460905 10.1038/nature11003 PMC3320027

[23] Drake JM, Paull EO, Graham NA, Lee JK, Smith BA, Titz B, et al Phosphoproteome Integration Reveals Patient-Specific Networks in Prostate Cancer. Cell. Elsevier; 2016;166: 1041–1054. doi: 10.1016/j.cell.2016.07.007 27499020 10.1016/j.cell.2016.07.007 PMC4985183

[24] Spagnolo F, Ghiorzo P, Queirolo P. Overcoming resistance to BRAF inhibition in BRAF-mutated metastatic melanoma. Oncotarget. 2014;5: 10206–10221. doi: 10.18632/oncotarget.2602 25344914 10.18632/oncotarget.2602 PMC4279367

