# Supplementary figures and images for: Network propagation in the cytoscape cyberinfrastructure
Source: PLoS Comput Biol. 2017 Oct 12;13(10):e1005598. doi: 10.1371/journal.pcbi.1005598 (PMC5638226; doi:10.1371/journal.pcbi.1005598)

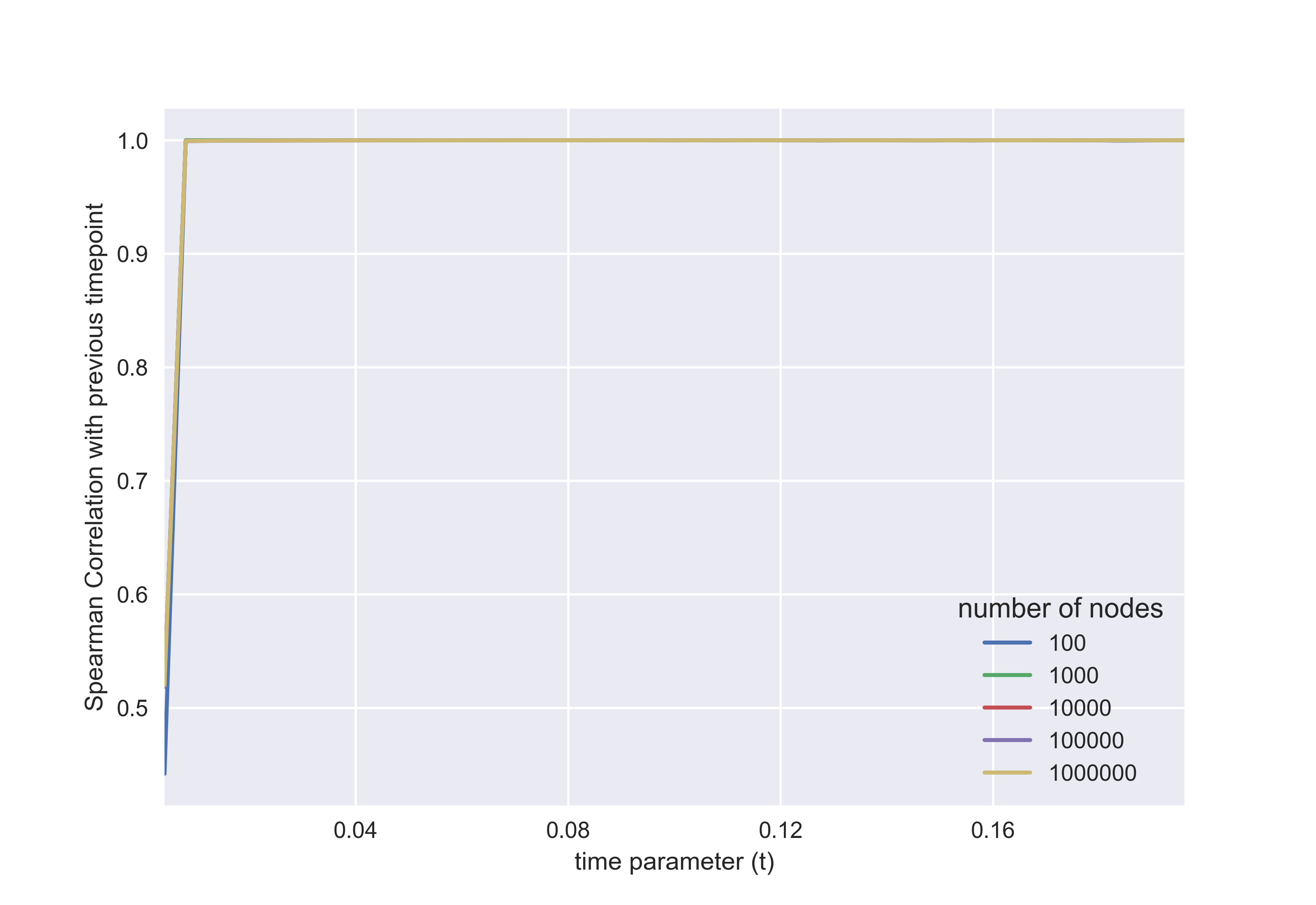

Supplement: S1 Fig — Effect of different choices of time parameter t on the convergence of queries on different sizes of random networks. As a measure of convergence, we track the Spearman correlation between adjacent time points. Note that near the default parameter of t = 0.1, queries on all sizes of network have converged. (PNG) [file pcbi.1005598.s001.png]

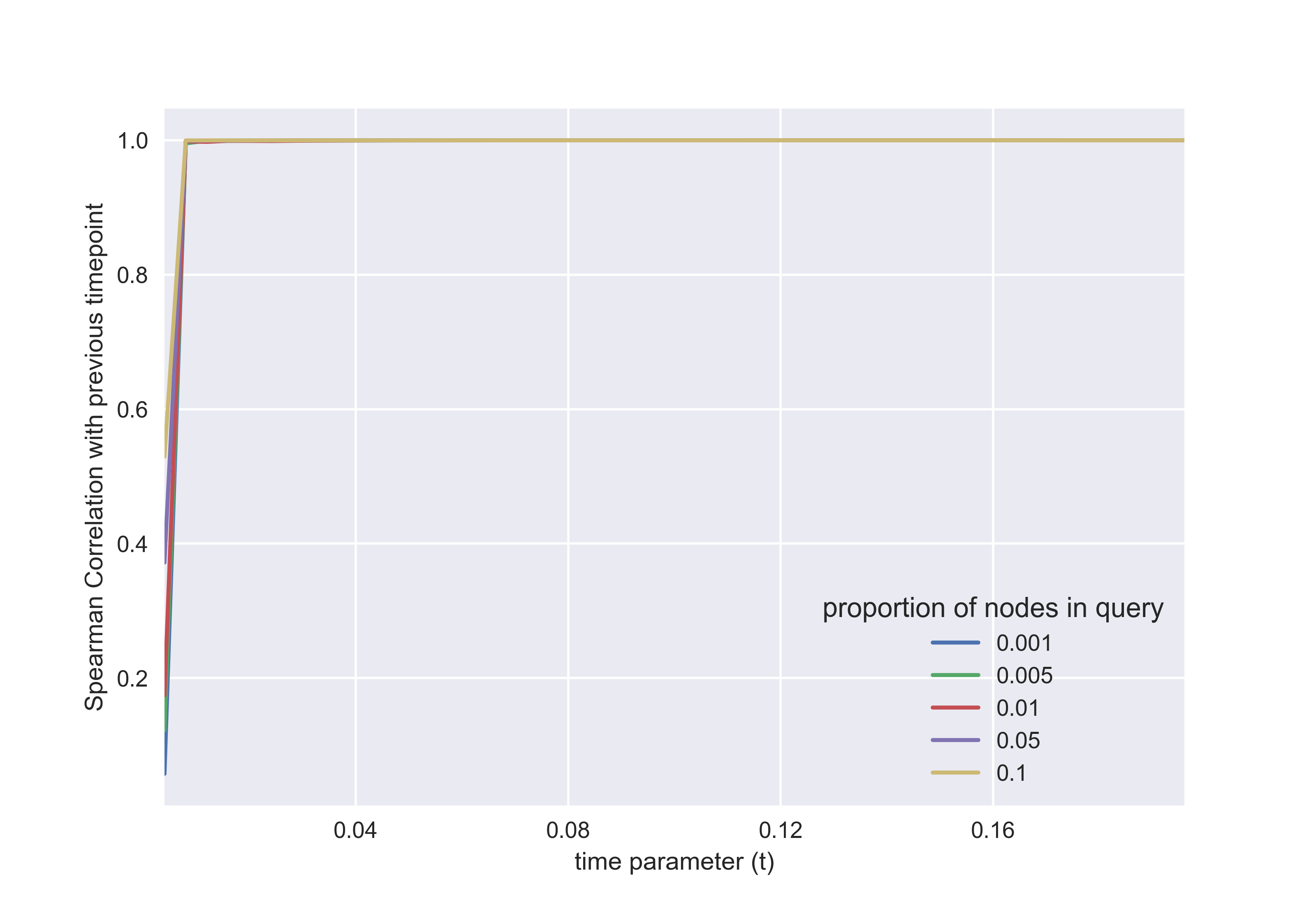

Supplement: S2 Fig — Effect of different choices of time parameter t on the convergence of queries of different proportions of a 10,000 node random network. As a measure of convergence, we track the Spearman correlation between adjacent time points. Note that near the default parameter of t = 0.1, all sizes of query have converged. (PNG) [file pcbi.1005598.s002.png]
